# Challenges in Extubation: A Case of a Non-removable Tracheal Tube Following Surgery

**DOI:** 10.7759/cureus.52787

**Published:** 2024-01-23

**Authors:** Rita Silva, Tania Carvalho, Maria João Tarroso, Helder Cardoso

**Affiliations:** 1 Anesthesiology, Centro Hospitalar Tâmega e Sousa, EPE, Penafiel, PRT

**Keywords:** tracheal cuff rupture, lodged tube, endotracheal tube cuff, airway extubation, difficult airway management

## Abstract

This report details a challenging case of difficult extubation due to a lodged tracheal tube following surgery, presenting an unexpected and complex clinical situation. An inspection of the airway using videolaryngoscopy revealed an over-inflated cuff beneath the vocal cords. Initial efforts to deflate the cuff with various methods were unsuccessful. The situation was ultimately resolved through the intervention of an otolaryngology surgeon. This case not only reviews various mechanisms of difficult endotracheal tube removal reported in the literature, but also underscores the potential for serious complications and highlights the critical role of multidisciplinary collaboration in managing extubation challenges. Additionally, our manuscript discusses alternative strategies that can be employed in scenarios where an otolaryngology surgeon is not available, offering practical guidance for anesthesiologists confronted with similar situations.

## Introduction

Extubation, the process of removing a tracheal tube, is a crucial step following general anesthesia [1]. The American Society of Anesthesiologists defines difficult extubation as 'the loss of airway patency and adequate ventilation after the removal of a tracheal tube or supraglottic airway from a patient with a known or suspected difficult airway' [2]. While significant attention is given to predicting and managing difficult airways, the occurrence of a tracheal tube becoming lodged and non-removable at the end of surgery is a less documented but potentially severe complication. The frequency of this complication is not well-established; yet, its occurrence can lead to critical situations, even in patients without prior indicators of a difficult airway. The factors leading to the problematic removal of a tracheal tube are varied, and identifying an effective solution is not always straightforward. This case report details an instance of challenging extubation resulting from a tracheal tube resistant to extraction post-surgery. We will explore some mechanisms of difficult endotracheal tube removal already described in the literature and discuss effective management strategies for such an unexpected situation. Furthermore, recognizing the importance of preparedness in clinical practice, especially when multidisciplinary assistance is not available, this report also highlights alternative methods and approaches that can be employed in similar challenging scenarios. Our aim is to contribute to a broader understanding and better preparedness for this rare but significant event.

## Case presentation

**Figure 1:**
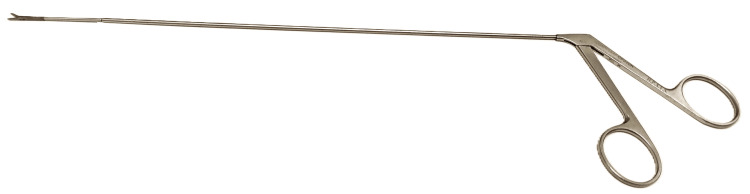
Laryngeal polypectomy scissors.

A 67-year-old woman with diabetes, poorly controlled hypertension, and obesity (BMI of 32 kg/m²) underwent total hip replacement surgery under general anesthesia. Despite her obesity, there were no other predictors of a difficult airway. During intubation, a Cormack-Lehane score of 2A was visualized, and intubation was successfully achieved on the first attempt using a Macintosh blade number four and a 7.5 mm internal diameter tracheal tube. The cuff was inflated to a pressure of 30 mmHg, and the tube was secured to the patient's face with adhesive tape. The surgery lasted approximately 2.5 hours without any notable complications. At the conclusion of the procedure, the neuromuscular blockade was fully reversed, and the patient was awakened. Despite completely deflating the pilot cuff and removing the adhesive tapes, noticeable resistance was encountered when attempting to remove the tube, leading to the decision to abort the extubation. The patient, now awake, began exhibiting signs of agitation, necessitating the re-induction of general anesthesia and the request for assistance from another anesthesiologist. A subsequent videolaryngoscopy was performed to inspect the airway, revealing an over-inflated cuff positioned beneath the vocal cords, despite the complete deflation of the pilot balloon. Efforts to re-inflate and deflate the cuff to alleviate potential valve blockage proved unsuccessful. Cutting the pilot balloon tubing was also attempted but did not resolve the issue. A direct puncture of the cuff with a 25G spinal needle under direct laryngoscopy was considered but ultimately abandoned due to challenges in safely guiding the needle and concerns about potential airway injury. The impasse necessitated the involvement of an otolaryngologist surgeon, who chose to rupture the cuff under direct rigid suspension laryngoscopy using straight scissors typically used in laryngeal polypectomies (Figure 1). Notably, the cuff's rupture was not as immediate as anticipated due to difficulties the scissors had in gripping the cuff. Following this intervention, the patient was closely monitored in the post-anesthetic unit. No airway-related complications were observed, and the patient's recovery proceeded uneventfully thereafter.

## Discussion

Extubation, a critical procedure following general anesthesia, often encounters challenges. The failure to remove a firmly fixed tracheal tube can lead to significant complications. Numerous cases in medical literature document challenges encountered during tracheal tube extubation, with various mechanisms and solutions identified:

1) A cuff that did not deflate due to kinking in the pilot tube, caused by tapes securing the tracheal tube to the patient's face, was resolved in different ways. These included removing the tapes causing the kink [3], extubating with the cuff still inflated [4], cutting the pilot line [5], aspirating the pilot line distally to the obstruction with a 16G needle [6], directly puncturing the cuff with a 22G spinal needle through the vocal cords [7], and puncturing the cuff through the cricoid membrane [8]. 2) Abnormal folds in a fully deflated cuff, preventing tracheal tube removal in a narrow airway, were addressed by subtly insufflating the cuff to undo the folds and facilitate tube removal [9]. 3) A tracheal tube that was too wide and forcefully introduced into a patient's airway required rotation of the tube 180 degrees for extraction [10]. Subglottic stenosis in a patient with a previous tracheostomy, following a five-hour surgery, led to airway swelling. After 48 hours of waiting for the swelling to subside, surgical cricoid division was necessary to remove the tracheal tube [11]. In a case of congenital subglottic stenosis, successful extubation was achieved with a "strong pulling force" [12]. 5) A subglottic pedicled mass, impacting and blocking tube removal, was solved with the assistance of an otolaryngologist who manipulated the mass [13]. 6) A barb accidentally made in the tracheal tube during maxillofacial surgery was resolved through gentle manipulation of the tracheal tube during extraction [14]. 7) An entangled pilot tube with a nasogastric tube, preventing extubation, was resolved by cutting the pilot line [15]. 8) A Kirshner wire, inserted between the nasotracheal tube and its pilot line during maxillofacial surgery, led to the tube's fixation. Cutting the pilot line resolved this [16]. 9) Transfixation of a nasotracheal tube by a Kirshner wire during maxillofacial surgery was revealed through radiographic evaluation and bronchoscopy [17]. 10) Transfixation of the tracheal tube by a surgical screw during maxillofacial surgery was identified by bronchoscopy and resolved surgically [18]. 11) Fixation of the tube by sutures during thoracic surgery led to a fatal outcome caused by laceration of the pulmonary artery [19]. A tracheal tube sutured to a bronchus was identified after blood was observed in the tube post-extubation, leading to immediate repair of the laceration without major consequences [19]. 12) An unknown mechanism caused a difficult double-lumen tube removal after thoracic surgery, leading to a 7 cm longitudinal laceration in the trachea's posterior wall [20].

**Figure 2:**
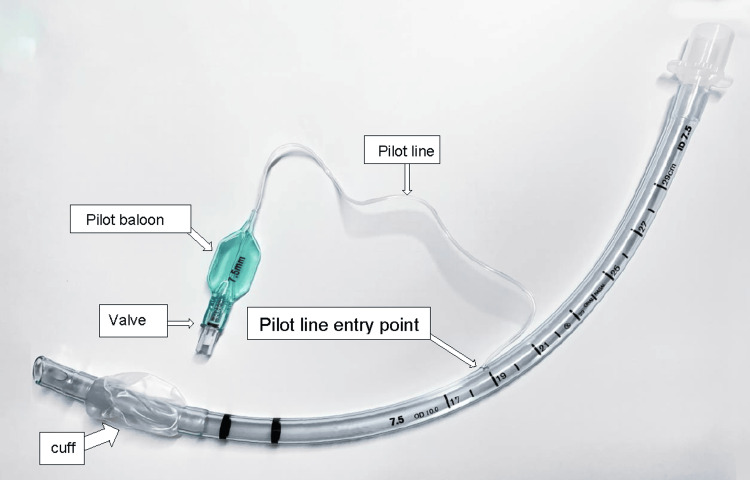
Tracheal tube composition. Photograph and illustration provided by the author Helder Cardoso.

Complications from the difficult removal of a securely fixed tracheal tube can be serious and varied. They include delayed extubation, patient awareness, agitation, inadvertent forceful removal by the patient, airway trauma, airway obstruction, disruption of surgical sutures, pneumothorax, and potentially life-threatening hemorrhage. These risks are notably elevated when managing a previously identified difficult airway, underscoring the crucial importance of exercising caution and making timely decisions during the extubation process. In these challenging situations, it is important for anesthesiologists to be familiarized with possible causes of tube fixation and the various strategies for addressing them. Understanding the composition of a tracheal tube is also important (Figure 2).

**Figure 3:**
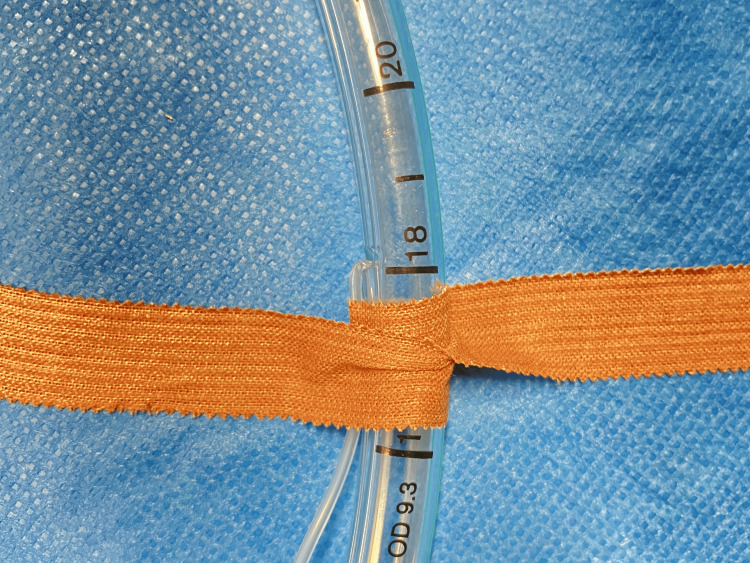
Illustration of adhesive tapes on a tracheal tube causing kinking of the pilot line. Photograph provided by the author Helder Cardoso.

In our specific case, a detailed examination of the tube post-removal revealed that the kinking was caused by fixing tapes. These tapes, though initially removed from the patient's face, were inadvertently left wrapped around the tube, precisely over the pilot line (Figure 3).

Such an oversight should not have occurred. We openly acknowledge this mistake, recognizing that sharing this information adheres to the professional commitment to learning from avoidable errors. Reporting these incidents is essential; it raises awareness and contributes to the prevention of similar occurrences in the future. In reflecting on the positive aspects of our clinical management, we emphasize the judicious decision to reinduce general anesthesia. This step was crucial in preventing harm due to the patient's agitation and allowed for a careful inspection of the airway, leading to the identification of an inflated cuff as the cause of resistance to removal. Additionally, we highlight the importance of promptly seeking assistance from another anesthesiologist and the decisive action to escalate the level of help by involving multidisciplinary support. Notably, the involvement of an otolaryngology surgeon was key. He safely ruptured the cuff under direct suspension laryngoscopy using scissors commonly employed in laryngeal polypectomies. It is important to note that the cuff's rupture by the surgeon was neither easy nor as swift as expected. This observation suggests that attempting to rupture the cuff by other means, such as using a spinal needle by the anesthesiologist through conventional direct laryngoscopy, might not be as straightforward as one might initially anticipate. However, the mechanisms and solutions discussed earlier in this manuscript provide alternative and potentially vital strategies that could be crucial, especially in scenarios where an otolaryngology surgeon is not available for assistance.

## Conclusions

To mitigate the risk of complications during the extubation process, strict adherence to established extubation guidelines is essential. Forcibly removing a tube that resists extraction should never be attempted, as the consequences could be significant, even catastrophic. Key measures include ensuring that the pilot tube is protected from compressions and kinks during the fixation of the tracheal tube, and inspecting the pilot line to ensure it is free from obstructions if an undeflatable cuff is suspected. Additionally, the prompt involvement of multidisciplinary support, specifically from an otolaryngology surgeon, can be crucial in safely managing these complex situations.
